# The respiratory microbiome: dynamics from health to disease

**DOI:** 10.1093/femsec/fiag058

**Published:** 2026-06-22

**Authors:** Wenxin Lin, Zheng Sun, Jingxian Chen, Shenghai Huang

**Affiliations:** School of Life Sciences, Anhui Medical University, Hefei 230032, Anhui Province, China; School of Life Sciences, Anhui Medical University, Hefei 230032, Anhui Province, China; Columbia University Department of Pediatrics, NY 10032, United States; School of Life Sciences, Anhui Medical University, Hefei 230032, Anhui Province, China; Department of Microbiology, The Institute of Clinical Virology, School of Basic Medical Sciences, Anhui Medical University, Hefei 230032, Anhui Province, China; Anhui Public Health Clinical Center, the First Affiliated Hospital of Anhui Medical University, Hefei 230012, Anhui Province, China

**Keywords:** respiratory microbiome, airway ecology, gut–lung axis, immune regulation, dysbiosis, multi-omics

## Abstract

Research on the respiratory microbiome has moved beyond the sterile-lung paradigm, but disease-associated microbial patterns are still often described as static signatures. In this mini-review, we synthesize current evidence within a dynamic state-transition framework in which respiratory microbial communities are shaped by microbial immigration, elimination, local growth conditions, and host inflammatory tone. This framework traces the respiratory microbiome from early-life assembly and homeostatic maintenance to perturbation, recovery, or persistence in alternative ecological states. We discuss how barrier integrity, mucociliary clearance, mucus and nutrient landscapes, inflammatory feedback, microbial metabolites, and the gut–lung axis regulate microbial stability and disease susceptibility. Across asthma, chronic obstructive pulmonary disease, cystic fibrosis, bronchiectasis, and respiratory infection, dysbiosis is interpreted not as a set of disease-specific taxa, but as a context-dependent outcome of shared ecological mechanisms. We also highlight methodological and translational priorities, including contamination control in low-biomass samples, longitudinal sampling, multi-omics integration, spatial host profiling, and cautious interpretation of association versus causality. Viewing the respiratory microbiome as an ecological system in motion may better connect microbial dynamics with disease heterogeneity, risk stratification, and future microbiome-directed interventions.

## Teaser

The respiratory microbiome is a living, changing ecosystem-not a static disease signature-shaped by immunity, environment, and ecology.

## Introduction

The human respiratory tract, once considered sterile below the larynx, is now recognized as a complex microbial ecosystem that interacts continuously with the airway epithelium and immune system (Dickson and Huffnagle 2015, Dickson et al. 2015, Man et al. 2017, Natalini et al. 2023). Respiratory microbial communities contribute to baseline immune education, epithelial barrier maintenance, and colonization resistance against invading pathogens.

Although the respiratory microbiome is now routinely associated with asthma, chronic obstructive pulmonary disease (COPD), cystic fibrosis (CF), pneumonia, and other airway disorders, many studies still describe these relationships as static dysbiosis signatures captured at a single time point. Such descriptions are useful but incomplete (Dickson et al. 2014, Natalini et al. 2023, Perez-Cobas et al. 2023). From an ecological perspective, respiratory health is better understood as a moving equilibrium shaped by microbial immigration, microbial elimination, and local growth conditions, all of which vary across airway niches and across the lifespan.

**Figure 1:**
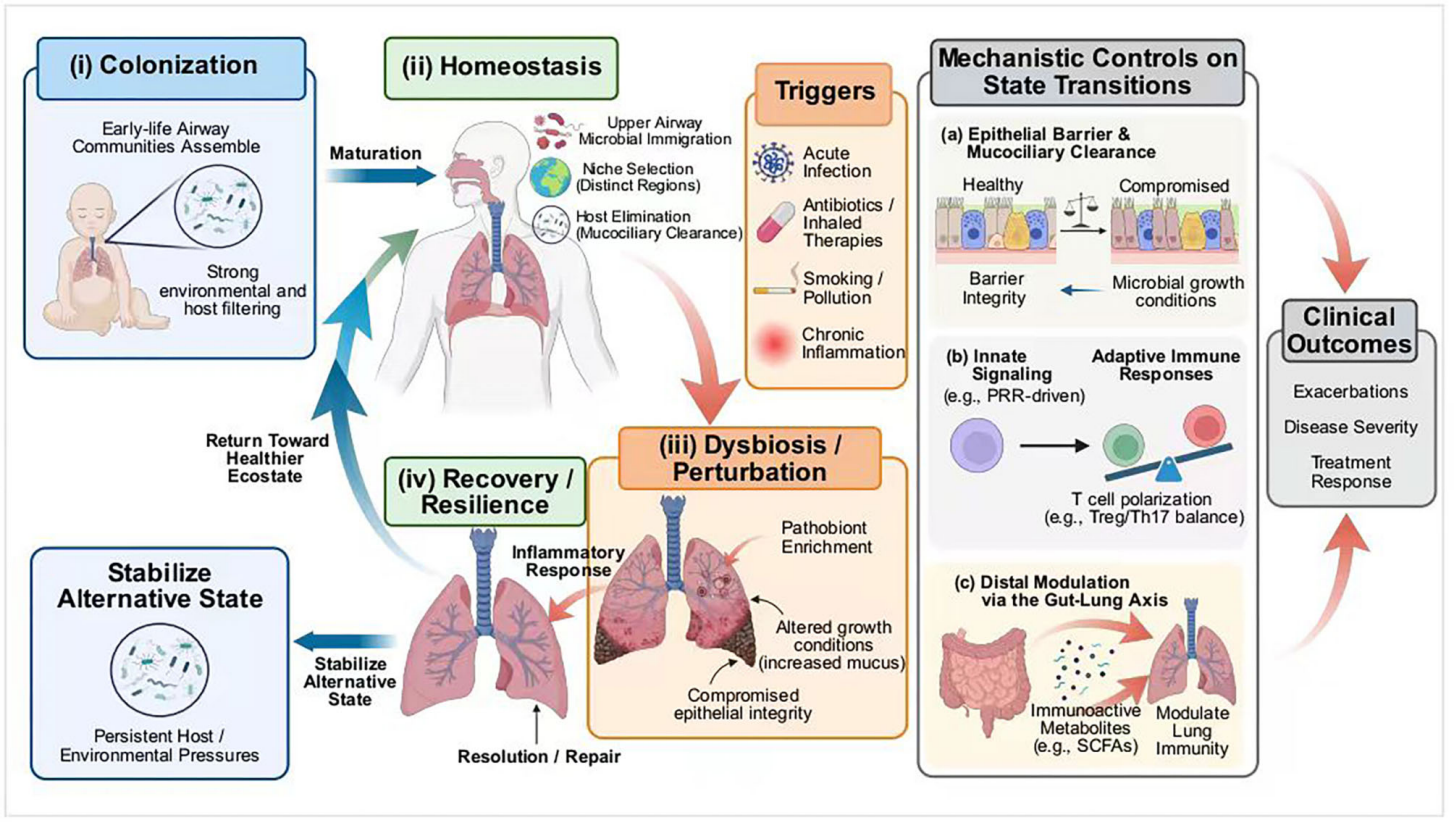
Ecological state-transition framework for the respiratory microbiome.

This review therefore adopts a dynamic ecological perspective. Rather than cataloguing respiratory diseases one by one, we organize current evidence around temporal state transitions and shared mechanistic themes. We ask how respiratory communities are spatially structured, how they participate in host immune regulation, how they shift across colonization, homeostasis, perturbation, recovery, and chronic persistence, and which methodological advances are required to move from association toward mechanism and translation. This perspective allows disease-associated microbial patterns to be interpreted through shared mechanisms, including altered barrier function, mucociliary clearance, nutrient and mucus availability, inflammatory feedback, and reduced ecological resilience. The proposed framework is summarized in Fig. 1 and serves as the conceptual scaffold for the sections that follow (Natalini et al. 2023, Perez-Cobas et al. 2023, Odendaal et al. 2024, Ozcam and Lynch 2024).

Early-life assembly feeds into a homeostatic state maintained by balanced microbial immigration, elimination, and local growth conditions. Infection, pollution, smoking, antibiotics, inhaled therapies, and structural lung disease can push communities toward perturbation or dysbiosis. Barrier integrity, mucociliary clearance, inflammatory signaling, microbial metabolites, and the gut–airway axis influence whether the system recovers, remains unstable, or settles into a chronic alternative state associated with recurrent exacerbation and disease progression.

## Spatial topography and ecological gradients of the respiratory microbiome

The respiratory tract is best viewed as a continuous ecological gradient rather than as two disconnected compartments (Dickson et al. 2014, 2015). The upper respiratory tract (URT) acts as a major reservoir of organisms, whereas the lower respiratory tract (LRT) contains a lower-biomass community that is continually shaped by microaspiration, inhalation, mucociliary clearance, coughing, and immune surveillance. In healthy hosts, the LRT is therefore not colonized in the same manner as the gut; rather, it reflects a dynamic balance between ongoing microbial input and ongoing removal.

Moving from the nasal cavity to the alveoli, the physical environment changes substantially, with shifts in temperature, oxygen tension, mucus composition, nutrient availability, and immune pressure. These local ecological filters help determine the spatial topography of the respiratory microbiome. Consequently, while the URT harbors diverse communities that can seed distal sites, the LRT is characterized by lower total biomass and stronger dependence on immigration–elimination balance rather than dense resident colonization. Recent work also suggests that airway epithelial structure and host physiology shape localized microbial niches, reinforcing the view that respiratory microbial communities are integrated into the host anatomical and functional landscape. Population-scale data further show that airway biogeography is modified by age, season, social interaction, smoking, alcohol use, antibiotic exposure, and other host–environmental factors, with many associations being strongly site- and age-dependent (Odendaal et al. 2024).

This interpretation is consistent with the adapted island model of lung biogeography and with later work emphasizing that low-biomass respiratory communities are especially sensitive to host and environmental filtering. Table 1 summarizes recurring ecological features across major airway sites.

**Table 1:** Recurring ecological features of major respiratory niches.

Anatomical region	Microenvironment and ecological drivers	Common taxa in health	Typical dysbiotic shift
Nasal cavity and nasopharynx (URT)	High airflow, lower temperature, strong environmental exposure, prominent mucociliary filtering.	*Corynebacterium, Dolosigranulum, Moraxella, Staphylococcus, Streptococcus*	Seasonal, infectious, or inflammatory shifts can favor pathogen-associated profiles and reduced stability.
Oropharynx (URT)	Shared mucosal interface with the oral cavity, saliva flow, mixed oxygen conditions, frequent immigration to distal sites.	*Streptococcus, Neisseria, Prevotella, Veillonella, Haemophilus*	Community structure can shift with smoking, antibiotics, immunosuppression, and oral-to-airway spillover.
Trachea and bronchi (conducting airways)	Continuous immigration from the URT, mucociliary escalator, antimicrobial peptides, intermediate oxygen and nutrient availability.	Low-biomass communities often resembling oral and upper-airway source taxa, including *Prevotella, Veillonella*, and *Streptococcus*	Chronic inflammation and impaired clearance may favor pathobiont enrichment and recurrent exacerbation-associated instability.
Alveolar compartment (distal LRT)	Lowest biomass, strong immune filtering, surfactant-rich environment, intense dependence on immigration-elimination balance.	Transient, low-biomass communities largely reflecting upstream sources rather than dense resident colonization	Barrier disruption, pneumonia, and severe structural disease can permit persistent pathogen expansion.

**Note:** The table summarizes recurring ecological patterns rather than fixed taxonomic rules; exact community composition varies with age, sampling method, disease context, and recent treatment exposure.

## Bidirectional host-microbiome immune cross-talk

The respiratory microbiome both shapes and is shaped by host immunity. This bidirectional cross-talk contributes to mucosal tolerance, baseline immune readiness, and pathogen defense (Man et al. 2017) (Huffnagle et al. 2017).

Host–microbiome interactions in the respiratory tract are best interpreted as coupled ecological feedback loops rather than isolated immune signals. Changes in microbial community structure can alter epithelial barrier function, mucus composition, metabolite availability, and innate immune tone; these host responses, in turn, modify oxygen tension, nutrient gradients, antimicrobial pressure, and microbial clearance. Dysbiosis may therefore be both a consequence and a driver of altered airway ecology. This feedback-loop perspective helps explain why similar microbial signatures may have different clinical implications depending on host inflammatory status, airway structure, recent treatment exposure, and the capacity for ecological recovery (Dickson and Huffnagle 2015).

### Local immune priming and colonization resistance

Commensal microbes contribute to innate immune priming by calibrating the activation thresholds of alveolar macrophages and dendritic cells through pattern-recognition pathways (Medzhitov and Janeway Jr 2000, Rosenberger and Finlay 2003).

This low-level stimulation helps maintain immune readiness and supports antimicrobial peptide production. At the same time, niche occupation and nutrient competition by resident microbes contribute to colonization resistance, limiting the attachment and expansion of exogenous pathogens (Natalini et al. 2023).

Loss of colonization resistance is not merely a taxonomic shift from “commensals” to “pathogens.” It represents a functional breakdown of ecological control. When commensal-derived immune priming, niche occupation, and nutrient competition are weakened, opportunistic taxa may expand in response to newly available adhesion sites, altered mucus-derived carbon sources, and reduced microbial competition. Their expansion can further stimulate epithelial and myeloid inflammatory pathways, creating a self-reinforcing cycle in which inflammation reshapes the niche and the reshaped niche sustains dysbiosis.

### The gut–lung axis and systemic immunomodulation

Airway homeostasis is also shaped by extra-respiratory microbial signals. Evidence from animal and human studies supports a bidirectional gut–airway axis in which gut-derived metabolites, microbial ligands, and immune programming influence distal airway immunity, while respiratory inflammation can reciprocally alter gut microbial activity (Ozcam and Lynch 2024, Marrella et al. 2024). Short-chain fatty acids (SCFAs) are the most frequently discussed mediators, with reported effects on macrophage responsiveness, epithelial function, and the balance between regulatory and effector T cell programs (Ozcam and Lynch 2024, Trompette et al. 2014). Integrated metagenome–metatranscriptome–metabolome profiling further suggests that common oral commensals contribute differentially to immunomodulatory metabolites across the respiratory tract, emphasizing that functional geography can matter as much as taxonomic overlap. At present, however, the translational promise of this axis exceeds the certainty of the clinical evidence, and microbiome-directed gut interventions should still be regarded as investigational.

The strength of evidence supporting the gut–lung axis also varies across experimental systems. Animal studies provide mechanistic support for metabolite-mediated immune regulation, whereas many human studies remain associative and are affected by diet, antibiotic exposure, disease severity, and medication use. Gut-derived signals are therefore best viewed as modulators of airway immune tone rather than as stand-alone therapeutic targets. Future studies should connect microbial metabolites, host immune phenotypes, and longitudinal clinical outcomes within the same cohorts before causal translation can be established.

## Temporal ecological transitions of the respiratory microbiome

The respiratory microbiome is not a static entity, but a fluid ecosystem governed by selective pressures that change across the host lifespan. To understand its role in health and disease, the field must move beyond static compositional snapshots toward temporal ecological transitions (Natalini et al. 2023, Dickson et al. 2014). Across the lifespan, the respiratory microbiome can be conceptualized as moving through several states: early assembly, homeostatic maintenance, perturbation or dysbiosis, and either recovery or persistence in an alternative stable state. These states should not be treated as fixed taxonomic categories; rather, they describe ecological positions along a continuum shaped by microbial input, elimination, local growth conditions, and host inflammatory tone. Recovery therefore depends not only on restoration of microbial diversity, but also on epithelial repair, clearance mechanisms, and immune tolerance.

### Initial colonization and developmental trajectories

Respiratory microbiome assembly begins around birth but is not determined by a single factor. It reflects maternal microbial transmission, delivery mode, feeding patterns, antibiotic exposure, early environmental contact, and host immune maturation. Longitudinal infant studies indicate that the upper respiratory microbiota develops through non-random succession and that delivery mode, feeding, crowding, and antibiotic exposure can influence this trajectory (Bosch et al. 2016, Bosch et al. 2017, Pattaroni et al. 2018, de Steenhuijsen Piters et al. 2020). Lower-airway data further suggest co-development between microbial colonization and immune maturation during the first year of life. These findings support a developmental-window model, but they should not be overinterpreted as deterministic: early microbial profiles may modify risk rather than irreversibly define later respiratory disease.

At the other end of the developmental spectrum, aging is associated with niche- and age-specific changes in respiratory microbial composition. These shifts likely reflect the combined effects of immune aging, cumulative exposure history, medication use, comorbidity, and altered mucociliary or epithelial function, rather than a uniform collapse of microbial diversity. Such age-related changes may help explain increased susceptibility to severe respiratory infection and chronic respiratory decline in older adults.

### Homeostasis under environmental and lifestyle pressures

Homeostasis is not a passive resting state but an actively maintained equilibrium under strong host and environmental filtering. Smoking, recurrent inflammatory injury, seasonality, social contact patterns, and medication exposure can all alter airway community composition, often in niche-specific ways. Air pollution has also been associated with shifts in respiratory microbial composition and diversity, although the taxa reported vary across populations, exposure types, sampling sites, and analytical pipelines (Arca-Lafuente et al. 2025, Odendaal et al. 2024). Across chronic airway disease cohorts, persistent exposure to smoke and inflammation is repeatedly associated with lower diversity and pathobiont enrichment, but the exact taxa implicated vary by population, sampling method, and disease phenotype. Evidence from adult upper-airway studies similarly suggests pollution-associated compositional changes, while highlighting substantial heterogeneity in sample processing, sequencing workflows, and confounder control.

Seasonal variation further challenges this equilibrium. Peaks in respiratory viral infection can disrupt the epithelial barrier, transiently deplete protective commensals, and reduce host tolerance to secondary bacterial infection. Systemic lifestyle factors, particularly diet, may also modify airway immunity through the gut–lung axis. High-fiber diets support gut microbial production of short-chain fatty acids, which can circulate to the lungs and modulate inflammatory tone, illustrating how distal microbial ecosystems may provide background support for respiratory immune homeostasis.

### Perturbation and dysbiosis: acute infection, antibiotics, and inhaled therapies

Acute infections can rapidly shift airway ecosystems by disrupting epithelial integrity, altering mucus composition, and weakening colonization resistance. During SARS-CoV-2 infection, low-diversity, pathobiont-enriched upper-airway communities have been associated with worse clinical trajectories and stronger inflammatory signatures, illustrating how infection can amplify ecological instability (Xie et al. 2024, Marrella et al. 2024). Antibiotics remain essential for the management of many acute infections, but they can also deplete commensal taxa and open ecological space for opportunistic expansion.

Therapeutic perturbation is not limited to antibiotics. In COPD, emerging evidence indicates that inhaled corticosteroid (ICS)-containing regimens can reshape airway microbiome structure, although the magnitude and direction of these effects appear to depend on the specific formulation, treatment context, and baseline host state (Yip et al. 2022, Richardson et al. 2025, Luo et al. 2024).

These findings support the inclusion of inhaled therapies among the ecological triggers of airway state transitions.

### Ecological resilience and microbiome restoration

Following perturbation, some individuals return toward a healthier ecological state through epithelial repair, restoration of clearance mechanisms, and immune recalibration. Others do not. When mucus stasis, chronic inflammation, or structural damage persist, airway communities may stabilize in an alternative state that differs from the pre-perturbation baseline and is more permissive to recurrent exacerbation. In this context, resilience is more informative than any single diversity metric measured at one time point. Longitudinal COPD studies and large-scale network analyses support this view, showing incomplete post-exacerbation recovery and disturbed microbial interactomes even outside overt exacerbation events.

Non-antibiotic microbiome-directed approaches, including nutritional modulation, probiotics, and targeted ecological restoration strategies, are conceptually attractive but remain insufficiently validated for routine respiratory disease management. The stronger conclusion at present is not that these interventions reliably accelerate recovery, but that they illustrate a translational hypothesis: successful interventions may need to restore colonization resistance, epithelial repair, and immune recalibration simultaneously rather than simply increase microbial diversity.

## Shared mechanistic themes across disease contexts

The disease contexts below are discussed not as separate catalogues of taxa, but as repeated examples of shared ecological failure. Across asthma, COPD, CF, bronchiectasis, pneumonia, and viral infection, clinically relevant dysbiosis often reflects convergent processes: inflammatory selection, barrier disruption, altered nutrient availability, impaired clearance, structural remodeling, and incomplete recovery after perturbation.

### Inflammatory dysbiosis and immune dysregulation

Across chronic respiratory disease, dysbiosis is often linked to altered immune tone rather than to a single pathogenic taxon. In asthma, reduced commensal diversity and enrichment of potential pathogens have been associated with phenotype- and endotype-specific immune dysregulation, including both type 2-high and non-type 2 inflammatory patterns depending on host context. Recent sputum multi-omics work further supports metabolically distinct asthma endotypes linked to Streptococcus-dominant dysbiosis rather than a universal asthma signature (Chun et al. 2020, Kim and Bunyavanich 2025, Liu et al. 2025). In COPD, microbiome shifts similarly interact with smoking history, exacerbation frequency, corticosteroid exposure, and inflammatory endotype (Yip et al. 2022, Luo et al. 2024).

Together, these findings suggest that inflammatory dysbiosis is better interpreted as a context-dependent interaction between host immune tone, treatment exposure, and ecological niche selection than as a disease-specific microbial signature.

### Barrier disruption and loss of colonization resistance

A second recurring theme is barrier failure. Viral infection, toxic exposure, and chronic inflammation alter mucociliary clearance, epithelial integrity, and nutrient availability, thereby changing local growth conditions and reducing colonization resistance. These changes create ecological windows for secondary bacterial expansion and help explain why acute infection can precipitate both short-term worsening and longer-term microbiome instability. COVID-19 provides a recent illustration of this process, but the same principle is relevant across influenza, bacterial pneumonia, and chronic airway disease. Mechanistically, barrier failure changes the ecological rules of the airway by exposing adhesion sites, increasing leakage of host-derived nutrients, altering mucus architecture, and weakening physical clearance. These changes can select for opportunistic organisms even when the initiating trigger is viral, toxic, or inflammatory rather than bacterial. In this way, barrier disruption provides a common ecological entry point through which transient microbial fluctuations can become clinically relevant dysbiosis.

### Structural remodeling and chronic persistence

CF provides an archetypal example of entrenched host–microbiome feedback. Abnormal ion transport, thick mucus, repeated antibiotic exposure, and structurally altered airways favor chronic persistence of specialized communities, including biofilm-forming pathogens such as Pseudomonas aeruginosa (Thornton et al. 2022, Borisova et al. 2025, Cauwenberghs et al. 2024). Even in the era of CFTR modulators, microbiome-directed strategies are best viewed as adjuncts rather than replacements for standard therapy, underscoring how difficult it is to reverse an already stabilized disease-associated ecosystem. Related patterns are increasingly recognized in bronchiectasis, where pathogen-dominant communities are linked to structural disease burden, neutrophilic inflammation, and exacerbation risk (Mac Aogáin et al. 2024, Chen et al. 2024). These examples illustrate why microbiome change in chronic lung disease should be interpreted as a host-structured ecosystem: microbial persistence is sustained by mucus viscosity, oxygen gradients, antibiotic pressure, immune evasion, and biofilm physiology rather than by pathogen presence alone. Structural remodeling therefore represents a mechanism by which temporary dysbiosis can become spatially stabilized, making microbial persistence harder to reverse even when acute inflammation is treated.

### Ecological resilience and clinical severity

Exacerbations can be interpreted as failures of ecological resilience rather than as isolated clinical events. Exacerbation-prone states in chronic airway disease frequently show reduced diversity, repeated dominance by opportunistic taxa, and incomplete recovery after treatment. These patterns may eventually support risk stratification and treatment selection, but they are not yet mature enough to serve as routine clinical biomarkers. A resilience perspective helps reconcile apparently inconsistent cross-sectional findings, because a single sample may capture an acute perturbation, a treatment response, a recovery trajectory, or a stable chronic state. From this perspective, exacerbation-prone disease reflects not only repeated exposure to triggers but also a reduced capacity of the airway ecosystem to return to its previous homeostatic state.

## Methodological and translational priorities

### Low-biomass sampling, contamination, and confounding

The greatest methodological challenge in respiratory microbiome research remains the low-biomass nature of many airway samples, especially those from the LRT. Recent expert guidance on low-biomass microbiome studies emphasizes contamination prevention and reporting across sample collection, extraction, library preparation, and data analysis (Fierer et al. 2025, Odendaal et al. 2025). This issue is particularly important because reagent contaminants or upper-airway carry-over can overwhelm true biological signal in sparse samples.

Bioinformatic tools such as decontam can improve data quality by identifying probable contaminant sequences, but they cannot compensate for weak study design (Davis et al. 2018). Interpretation is further complicated by confounders such as antibiotic exposure, inhaled corticosteroid use, comorbidity burden, diet, geography, and sampling strategy, all of which can produce microbiome differences that are mistaken for disease effects. These factors may generate apparent disease-associated signatures even when the dominant signal reflects treatment history, disease severity, sampling site, or recent infection. Accordingly, future analyses should distinguish microbial abundance from relative abundance, include sensitivity analyses for contamination and medication exposure, and avoid interpreting cross-sectional associations as causal pathways.

### Standardization and longitudinal study design

Standardized collection and processing protocols are therefore essential. Methodological work on low-biomass respiratory profiling highlights the value of consistent sampling, bacterial load quantification, and explicitly described downstream workflows. Beyond technical harmonization, future studies should prioritize repeated sampling and richly annotated metadata so that investigators can distinguish transient fluctuations from genuine ecological transitions (Odendaal et al. 2024). Where feasible, paired oral, upper-airway, lower-airway, and gut sampling may also help clarify source–sink relationships within the wider respiratory ecosystem. Longitudinal sampling is especially important because the central biological question is often not whether a dysbiotic state exists, but whether the community returns toward baseline, shifts to a new stable state, or remains trapped in a recurrent inflammatory loop.

### From association to mechanism and intervention

Moving from association to mechanism will require combining taxonomic profiling with metagenomics, metatranscriptomics, metabolomics, host transcriptomics, and spatially resolved assays (Singh et al. 2022, Yan et al. 2022, Gao et al. 2023, Wong et al. 2025)

Recent perspectives in chronic airway disease argue that community ecology offers a stronger framework for this next phase than purely descriptive case-control designs. This transition is already becoming feasible: paired metagenome–metatranscriptome–metabolome analyses have shown that common respiratory commensals contribute differently to local metabolic niche formation across the tract, and asthma-focused multi-omics studies are beginning to resolve clinically relevant microbe–metabolite endotypes. What matters is not only who is present, but which functions are active, how host responses are organized in time and space, and whether perturbations resolve or persist. Emerging single-cell and spatial transcriptomic studies of airway tissue, together with methodological reviews that standardize spatial analytic workflows, are also providing a route to connect microbial states with epithelial, stromal, and immune programs at precise anatomical locations (Joulia et al. 2025). Important blind spots remain: fungi and viruses are increasingly recognized as part of the airway ecosystem, but the mycobiome and virome are still less well resolved than bacterial communities in most clinical cohorts. Multi-omics will strengthen inference only when microbial features are linked to host transcriptional programs, metabolite gradients, immune phenotypes, and clinical trajectories in the same samples or cohorts. Otherwise, adding omics layers risks producing parallel associations rather than mechanistic explanation.

Therapeutically, microbiome engineering remains promising but early (Ozcam and Lynch 2024, Cauwenberghs et al. 2024, Hern and Prindle 2025). Diet-based modulation, probiotics, bacteriophages, and inhaled microbiome-directed approaches are all under active discussion, yet none can currently be considered standard respiratory care. The next generation of studies should therefore link mechanism to intervention more explicitly, using longitudinal and functional endpoints rather than relying solely on alpha-diversity changes or taxonomic associations. A cautious translational pathway is needed, progressing from reproducible association to longitudinal prediction, mechanistic validation, interventional testing, and, only then, clinical implementation.

## Conclusions

The respiratory microbiome should be viewed as a dynamic ecological system rather than a static list of organisms associated with disease labels. A state-transition framework, spanning colonization, homeostasis, perturbation, recovery, and chronic alternative states, better captures how host factors, environmental exposures, infections, and therapies interact across time. At the same time, the field must remain methodologically cautious: low biomass, contamination, and confounding continue to limit causal inference. With stronger longitudinal designs and deeper functional profiling, ecological concepts such as resilience, disturbance, and recovery may become increasingly useful for respiratory precision medicine. The most informative future studies will be those that make temporal dynamics, spatial niche structure, host feedback, and methodological uncertainty explicit, rather than treating dysbiosis as a fixed disease label.
